# Knowledge, attitude and practice regarding intravenous medication errors prevention among ICU nurses and influencing factors: a multicenter, cross-sectional study

**DOI:** 10.3389/fmed.2025.1657459

**Published:** 2025-11-21

**Authors:** Jingui Huang, Yanhong Deng, Niuniu Chen, Jing Nie, Hongjie Xu, Yumei Shi

**Affiliations:** 1 Department of Medical Oncology, Chongqing University Cancer Hospital, Chongqing, China; 2 Radiation Oncology Center, Chongqing University Cancer Hospital, Chongqing, China; 3 Department of Intensive Care Unit, Yichang Central People’s Hospital, Yichang, Hubei, China; 4 Department of Intensive Care Unit, Chongqing University Cancer Hospital, Chongqing, China

**Keywords:** knowledge-attitude-practice, nurse, intensive care unit, intravenous medication errors, influencing factors

## Abstract

**Background and objective:**

In the intensive care unit (ICU), intravenous medication errors (MEs) pose serious risks to critically ill patients. ICU nurses, as the main executors of intravenous administration, are crucial in preventing MEs. Their knowledge, attitude, and practice (KAP) directly affect prevention outcomes. Identifying influencing factors helps develop targeted measures to ensure safer medication administration and reduce MEs. Thus, this research intends to examine the knowledge, attitude, and performance of ICU nurses concerning intravenous MEs and determine the factors influencing their KAP scores.

**Methods:**

Between December 2024 and May 2025, a multi-center cross-sectional study was carried out in nine hospitals located in Chongqing Municipality, Hubei Province, and Sichuan Province of China. An online survey was conducted among 447 ICU nurses via the QuestionnaireStar platform, using the General Information Questionnaire, the Knowledge, Attitude, and Behavior in Medication Errors Questionnaire (KAB-MEQ), and the Chinese Calling Scale. *T*-tests, one-way analysis of variance, and Pearson correlation analysis were performed to preliminarily identify influencing factors. Multiple linear regression analysis was used to explore independent factors associated with KAP scores.

**Results:**

Online data collection yielded 424 valid questionnaires, achieving a response rate of 94.9%. The total scores of KAB-MEQ were 82.01 ± 6.91. The proportion of participants with good knowledge, attitude and practice was 53.8, 84.4 and 66.3%, respectively. Multiple linear regression analysis indicated that education level, professional title, years of ICU experience, receipt of relevant training, and career calling independently affected the KAP scores of nurses.

**Conclusion:**

While ICU nurses generally held a positive attitude toward intravenous MEs, there was a noticeable deficiency in knowledge and a lack of standardization in practice. The results suggest that conducting refresher training, utilizing the exemplary roles of experienced nurses, and promoting a professional mission awareness may contribute to the improvement of ICU nurses’ knowledge, attitude, and practices in preventing intravenous MEs.

## Introduction

Drug safety is an important part of the safety management of medical institutions, and improving drug safety is an important goal to ensure global patient safety. In 2017, the World Health Organization (WHO) launched its third World Patient Safety Project, which focuses on medication safety (1). From 2023 to 2025, the Chinese Hospital Association has taken drug safety as one of the 10 safety goals for patients for three consecutive years, requiring the strengthening of drug management and drug safety training to reduce the occurrence of medication errors. The National Coordinating Council for Medication Error Reporting and Prevention delineates medication errors (MEs) as any preventable incident arising during the administration of medication, irrespective of whether it is executed by a healthcare provider, the patient, or a drug consumer, which has the potential to result in incorrect utilization or patient harm, including temporary or permanent disability, or fatality (2). It is reported that MEs account for about a quarter of all healthcare errors and have become the second most common factor that threatens patient safety (3). Studies have shown that the global incidence of MEs ranges from 2 to 14% (4). In the UK, the annual cost of MEs is around $500 million, equivalent to 42.0% of total health spending (5). In accordance with the WHO findings, the financial burden incurred by MEs amounts to $42 billion annually worldwide, accounting for 0.7% of the global health expenditure (1), resulting in huge economic losses.

Among different departments in hospitals, MEs in the intensive care unit (ICU) pose special challenges to hospital safety management. ICU is a department that provides medical monitoring and professional treatment and nursing for critical patients, and can provide timely and effective, systematic and high-quality intensive care and advanced life support for patients with multi-organ and multi-system failure or potentially high-risk factors (6, 7). The ICU presents a complex nursing environment, characterized by a high burden of work tasks and frequent interruptions among the staff, thereby predisposing the environment to an increased likelihood of MEs (8). Surveys indicate that the prevalence of MEs in ICU nurses in Ethiopia and South Korea was 40% (9) and 53.6% (10), respectively. Iranian scholar Marznaki et al. (11) found that the prevalence of MEs made by ICU nurses was 53.34% in the systematic review including fifteen studies. When ICU nurses perform nursing operations, they are vulnerable to interference from nursing colleagues, clinicians, changes in patient conditions, and alarms from medical equipment such as infusion pumps, ECG monitors, and ventilators, leading to nursing interruption and increasing the risk of MEs (12). Previous research has confirmed that work interruption is an important cause of MEs among nurses (13). The awful thing is, according to statistics, nursing interruptions occurred at a rate of 4.95–5.09 per hour in the ICU (12, 14). Therefore, it is necessary to attach great importance to MEs in ICU environment and strengthen the management and monitoring of MEs among nurses.

It is worth noting that among all kinds of MEs, intravenous MEs are particularly prominent (11, 15, 16), and the incidence is 5 times that of other ways of MEs (17). At present, intravenous fluid therapy has become one of the most common treatment interventions for ICU patients in emergency situations (18). A variety of highly vigilant drugs in the ICU, such as opioids, insulin, and vasoactive drugs, are mostly administered intravenously and have a higher risk of harm when MEs occur (19). In addition, due to medical conditions, ICU patients often need to establish multiple drug intravenous access at the same time, which also increases the risk of intravenous MEs. As the “final gatekeeper” between patients and intravenous MEs, nurses are the direct contacts and managers of drug treatment, involving the whole process of drug treatment, and play a crucial role in identifying and preventing MEs.

Nurses’ knowledge and attitude toward medication safety are pivotal in preventing intravenous MEs. First and foremost, adequate medication-related knowledge serves as the foundation for medication safety, and gaps in nurses’ knowledge can directly translate into adverse clinical outcomes. A variety of interconnected root causes contribute to MEs, with nurses’ grasp of drug-related knowledge and/or their access to relevant information serving as important determining elements (16, 20). A survey involving 262 Chinese nurses revealed that insufficient knowledge emerged as the primary barrier encountered by nurses during medication administration (21); A total of 141 nurses reported having committed MEs, with 28.4% of patients experiencing mild consequences (requiring close monitoring of vital signs, prolonged hospital stays) and 9.2% suffering severe consequences (permanent injury, need for cardiopulmonary resuscitation, coma, death) (21).

In addition, nursing is a profession that requires both technical and cognitive skills, and nurses’ positive attitudes during the medication administration process exert a profound impact on their practices. Positive safety attitudes, such as a willingness to report errors, strong error awareness, and a sense of professional responsibility, act as driving forces in shaping safe medication practices-bridging the gap between nurses’ knowledge of medication safety and actual clinical behaviors. Giannetta et al. (22) conducted a cross-sectional survey of 1,383 ICU nurses in 12 countries and found that the reasons for nurses’ medication errors (MEs) largely depended on their attitudes. Notably, a willingness to report MEs, a core component of positive safety attitudes, is often undermined by organizational barriers in the Chinese healthcare setting: prior studies targeting Chinese nurses have identified that they hesitate to report errors due to systemic tendencies to blame individual culprits rather than address broader workflow flaws (e.g., inadequate medication verification processes or unclear shift handover protocols), as well as fear of reprimand or punishment from administrators (23, 24). These findings collectively highlight that nurses’ attitudes toward medication safety are not isolated psychological states, but factors closely linked to the prevention and occurrence of MEs.

To fully address the complexity of intravenous MEs prevention, it is critical to recognize that actual medication practices-such as double checking, proper infusion preparation, and error reporting-serve as the vital bridge between nurses’ knowledge, attitudes, and improved patient safety outcomes. A nurse’s knowledge of intravenous drug compatibility and their strong error awareness or sense of professional responsibility can only translate into reduced MEs risks when manifested in consistent practices like verifying doses with a colleague or preparing infusions in adherence to sterile protocols (25). Without assessing these practices, the link between knowledge, attitudes, and patient safety remains theoretical. Relevant studies have indicated that MEs in clinical settings are associated with nurses’ unsafe medication practices, which are specifically manifested in omitting the double-check step, failing to strictly adhere to the 8R medication administration principle, and not implementing monitoring measures before and after medication administration (26, 27). Therefore, evaluating intravenous medication practices is equally critical. By examining how knowledge and attitudes shape actual practices, we can identify actionable targets to enhance patient safety in ICU.

The knowledge, attitudes, and practices (KAP) study represents a methodological approach employed to ascertain and quantify knowledge, attitudes, and practices of a specific population on a particular subject through questionnaires (28). At present, KAP research has been widely used in the public health field (29–31). In the context of heightened emphasis on patient safety and continuous improvement of medical quality, KAP studies play a crucial role in identifying the specific knowledge gaps, attitudes, and practices among nurses that could impact the effectiveness of response and prevention strategies on intravenous MEs. However, there has been a lack of relevant assessment tools in China until 2024, when Chen et al. (32) introduced and culturally adapted Knowledge, Attitude, and Behavior in Medication Errors Questionnaire (KAB-MEQ) in the ICU nurses. Consequently, there is currently a scarcity of research on the on the ICU nurses’ KAP on preventing intravenous MEs, potentially leading to inadequate strategies for preventing such errors and compromising patient safety. In this research topic, knowledge pertains to the ICU nurses’ understanding and mastery of information, guidelines, and professional expertise related to intravenous administration that is critical to reducing intravenous MEs. Attitudes encompass the perspectives of ICU nurses regarding the prevention of intravenous MEs, which entailed aspects such as safety training for intravenous medication, enhancing awareness of error prevention, assessing clinical skills, and timely reporting of MEs. Practice entails the concrete actions undertaken by ICU nurses in response to intravenous MEs, embodying the practical application of their acquired knowledge and attitudes within their routine professional activities.

Career calling is considered to have an incentive force, prosocial effects, and other practical significance (33). It refers to a strong passion toward a specific profession, recognition of one’s occupation, and a sense of delivering quality work (34). Research has demonstrated that career calling has a positive impact on achieving the significance of work. Individuals who exhibit a strong sense of calling are more likely to derive greater meaning from their professional and personal lives and demonstrate heightened levels of enthusiasm, belief, and dedication to their careers (35, 36). Based on this, we hypothesize that ICU nurses with a strong sense of career calling may exhibit higher levels of responsibility and professionalism, which could potentially improve their knowledge and attitudes toward MEs and reduce their incidence. However, there is currently a lack of research exploring this relationship in depth.

Based on the comprehensive review and analysis of the aforementioned literature, this study aims to utilize the Chinese version of KAB-MEQ in clinical practice to investigate the current status of knowledge, attitudes, and practices regarding intravenous MEs among ICU nurses in China. Furthermore, it seeks to explore the factors influencing these aspects, ultimately providing a theoretical basis for developing an intervention program to reduce intravenous MEs and improving the training system in this area.

## Materials and methods

### Research design

This is a multi-site, descriptive and cross-sectional study.

### Setting and subjects

From December 2024 to May 2025, a convenience sampling method was employed to select ICU nurses from 9 hospitals in Chongqing Municipality, Hubei Province, and Sichuan Province, China. The inclusion criteria were as follows: nurses must hold a Nursing Practice Certificate issued by the Ministry of Health of China and have at least 1 year of work experience in the ICU. Exclusion criteria included nurses who were on internship, further education, or rotation, as well as those who declined to participate or did not sign the electronic informed consent form. This study was conducted in accordance with the Declaration of Helsinki and obtained ethical approval from the hospital’s ethics review board (ID CZLS2024088-A). All participants provided voluntary consent to join the study.

### Sample size determination

In the context of multifactor regression analysis, the sample size should be calculated as 10 times the number of independent variables (37, 38). In this study, the Chinese Calling Scale (CCS) comprised 3 dimensions, while nurses’ general data included 12 variables, resulting in a total of 15 variables. To account for potential invalid questionnaires, the sample size was adjusted by increasing the original estimate by 20%, leading to a final calculated sample size of at least 187 participants.

### Instruments

#### General information questionnaire

The general information questionnaire was developed by the research team specifically for this study. The survey includes the following items: gender, age, educational level, hospital level, employment type, work experiences, type of ICU, technical title, position, whether they have received training related to intravenous medication administration.

#### Knowledge, Attitude, and Behavior in Medication Errors Questionnaire

The KAB-MEQ scale, developed by scholars Di Muzio et al. (39) in Rome, consists of three dimensions: knowledge (7 items), attitude (7 items), and behavior (5 items), totaling 19 items. The Cronbach’s *α* coefficient is 0.776. Responses of knowledge and behavior dimensions were quantified using a 5-point Likert scale (1 = strongly disagree to 5 = strongly agree), while the attitude dimension employs a 3-point Likert scale ranging from 1 (disagree) to 3 (agree). The total score ranges from 19 to 81, with higher scores indicating higher levels of nurses’ KAP regarding medication administration errors. Chen et al. (32) translated and revised the Chinese version of KAB-MEQ, consisting of 20 items, with a Cronbach’s *α* coefficient of 0.93, content validity of 0.98, and split-half reliability of 0.818. In this study, since the knowledge and practice dimensions were rated using a 5-point Likert scale and the attitude dimension was rated using a 3-point Likert scale, the 3-point Likert scale for the attitude dimension was converted to a 5-point Likert scale for calculation purposes. Therefore, the questionnaire score ranges from 20 to 100 points. According to the modified Bloom’s cut-off standards, the scores of each dimension and the total score for each participant were categorized into three levels: poor (<60%), moderate (60–79%), and good (≥80%). In the present study, the Cronbach’s *α* coefficient for the scale was 0.79.

#### Chinese Calling Scale

The CCS, developed by Zhang et al. (40), has been widely used for measuring career calling among Chinese nurses (33, 41). The scale consists of 11 items across three dimensions: Altruism, Guiding force, and Meaning and Purpose. Each item is scored using a Likert 5-point scale, ranging from 1 (strongly disagree) to 5 (strongly agree). The total score ranges from 11 to 55, with higher scores indicating a stronger sense of career calling. The Cronbach’s alpha for the overall scale is 0.84, with each of the three dimensions achieving a Cronbach’s alpha of 0.77. Cronbach’s alpha for this scale was 0.90 in the current study.

### Data collection

This study utilized electronic questionnaires with an attached consent page for data collection, employing the QuestionnaireStar platform to generate QR codes. Approval and collaboration from hospital and departmental leaders were obtained prior to the investigation. Questionnaires were distributed step by step, with standardized instructions provided within the questionnaire to explain the purpose, significance, and filling instructions of the survey. ICU nurses were required to complete and submit the questionnaires anonymously and independently. Each participating hospital assigned one research member to monitor the timely completion of questionnaires by respondents. All items were mandatory, and each electronic device was limited to one submission. Questionnaires were deemed invalid if all items were answered with the same option or the completion time was less than 200 s. The platform was opened on December 1, 2024, and closed on May 31, 2025.

### Data analysis

Statistical analyses were conducted using SPSS version 25.0 (IBM Corp., Armonk, NY). Categorical data were described using frequency and percentage. For continuous data, after confirming its normal distribution using the Kolmogorov–Smirnov test, they were described using mean and standard deviation (SD). The *t*-test or one-way analysis of variance (ANOVA) was used to compare the scores of knowledge, attitude, and behavior of MEs among ICU nurses with different characteristics. Pearson correlation analysis was applied to assess the association between career calling and MEs. And after confirming statistically significant results through *t*-test or ANOVA, multiple linear regression analysis was conducted to further explore the influencing factors. The significance level was set at *α* = 0.05 using a bilateral test.

To evaluate the assumptions of the regression model, diagnostic analyses were conducted. Potential outliers were identified using Cook’s distance, residual autocorrelation was assessed via the Durbin–Watson (DW) statistic, and multicollinearity was tested by calculating variance inflation factors (VIF).

## Results

### Residual autocorrelation, multicollinearity and outliers

Diagnostic analysis results validated the reliability of the regression model assumptions. Cook’s distance values ranged from 0.0000 to 0.0640 across the dataset, all below the threshold of <1.0, indicating no influential outliers. The DW statistic was 1.921, which fell within the acceptable range of 0–4 and closely approximated the ideal value of 2, confirming no residual autocorrelation. Additionally, VIF values for all predictor variables ranged from 1.075 to 4.733, all below the conservative threshold of 5, demonstrating the absence of significant multicollinearity among explanatory variables.

### Socio-demographic characteristics of respondents

A total of 447 responses were received. After excluding invalid questionnaires, 424 valid ones were ultimately collected, with an effective response rate of 94.9%. The median age of participants was 31.93 years (±6.68), with female participants accounting for 89.4% and male participants 10.6%. In terms of educational background, the majority of nurses (67.5%) had a bachelor’s degree, while only 4.2% held a postgraduate degree or higher. Regarding clinical experience, 65.8% of participants had between 1 and 10 years of ICU clinical work experience. The majority were employed in tertiary hospitals (71.0%) and primarily worked in general ICU units (67.5%). In terms of professional roles, 87.3% of participants were general nurses, with 49.3% holding the title of senior nurse. These characteristics are summarized in Table 1.

**Table 1:** Comparison of knowledge, attitude, and practice scores of ICU nurses with different characteristics for intravenous MEs.

Variables	Frequency (%)	Knowledge		Attitude		Practice		KAP	
		Mean ± SD	Test results	Mean ± SD	Test results	Mean ± SD	Test results	Mean ± SD	Test results
Age (years)									
≤30	207 (48.8)	29.31 ± 5.02	*F* = 4.339	31.30 ± 3.67	*F* = 4.430	19.60 ± 3.29	*F* = 7.157	80.21 ± 7.07	*F* = 13.383
31–40	157 (37.0)	30.13 ± 4.06	*p* = 0.005	32.08 ± 2.83	*p* = 0.004	20.63 ± 2.78	*p* < 0.001	82.85 ± 6.19	*p* < 0.001
41–50	48 (11.3)	31.73 ± 4.63		32.58 ± 2.02		21.50 ± 2.93		85.81 ± 5.95	
≥51	12 (2.9)	31.75 ± 5.29		33.67 ± 2.46		21.78 ± 3.34		86.92 ± 6.67	
Gender									
Male	45 (10.6)	30.02 ± 5.20	*t* = 1.603	32.69 ± 2.29	*t* = 7.517	20.58 ± 2.90	*t* = 0.555	83.29 ± 6.43	*t* = 0.052
Female	379 (89.4)	29.95 ± 4.65	*p* = 0.206	31.70 ± 3.31	*p* = 0.006	20.21 ± 3.17	*p* = 0.457	81.86 ± 6.96	*p* = 0.820
Marital status									
Married	310 (73.1)	30.15 ± 4.57	*t* = 2.449	31.88 ± 3.27	*t* = 0.691	20.20 ± 3.28	*t* = 6.574	82.22 ± 6.87	*t* = 0.112
Other (single, divorced)	114 (26.9)	29.44 ± 5.03	*p* = 0.118	31.60 ± 3.14	*p* = 0.406	20.40 ± 2.75	*p* = 0.011	81.44 ± 7.01	*p* = 0.738
Education level									
Junior college and below	120 (28.3)	28.00 ± 5.61	*F* = 15.602	31.58 ± 3.57	*F* = 1.300	19.55 ± 3.32	*F* = 8.223	79.13 ± 8.08	*F* = 16.992
Bachelor’s degree	286 (67.5)	30.76 ± 3.88	*p* < 0.001	31.83 ± 3.16	*p* = 0.274	20.41 ± 3.01	*p* < 0.001	82.99 ± 5.94	*p* < 0.001
Master’s degree and above	18 (4.2)	30.28 ± 6.24		32.89 ± 1.45		22.50 ± 2.73		85.67 ± 7.15	
Level of hospital									
Tertiary hospital	301 (71.0)	29.73 ± 4.67	*t* = −1.532	32.01 ± 2.88	*t* = 2.081	20.12 ± 3.09	*t* = −1.397	81.86 ± 6.36	*t* = −0.705
Secondary hospital	123 (29.0)	30.50 ± 4.76	*p* = 0.126	31.29 ± 3.94	*p* = 0.038	20.59 ± 3.25	*p* = 0.163	82.38 ± 8.12	*p* = 0.481
Type of hospital									
General hospital	332 (78.3)	29.96 ± 4.60	*t* = 0.464	31.82 ± 3.10	*t* = 0.622	20.15 ± 3.21	*t* = 1.094	81.93 ± 6.69	*t* = 1.786
Specialist hospital	92 (21.7)	29.93 ± 5.09	*p* = 0.496	31.74 ± 3.69	*p* = 0.431	20.62 ± 2.85	*p* = 0.296	82.29 ± 7.69	*p* = 0.182
Employment type									
Permanent staff	68 (16.0)	30.41 ± 4.88	*t* = 0.008	32.15 ± 2.92	*t* = 1.162	20.04 ± 3.72	*t* = 6.418	82.60 ± 6.71	*t* = 0.137
Contract labor	356 (84.0)	29.87 ± 4.67	*p* = 0.931	31.74 ± 3.29	*p* = 0.282	20.29 ± 3.02	*p* = 0.012	81.90 ± 6.95	*p* = 0.711
Type of ICU									
General ICU	286 (67.5)	30.20 ± 4.49	*t* = 1.527	31.77 ± 2.97	*t* = 1.140	20.27 ± 3.05	*t* = 2.920	82.23 ± 6.15	*t* = 0.954
Specialist ICU	138 (32.5)	29.46 ± 5.10	*p* = 0.128	31.87 ± 3.84	*p* = 0.286	20.22 ± 3.34	*p* = 0.088	81.55 ± 8.28	*p* = 0.340
Professional title									
Nurse	50 (11.8)	27.80 ± 5.51	*F* = 5.519	30.72 ± 5.32	*F* = 3.881	19.52 ± 3.40	*F* = 3.885	78.04 ± 9.06	*F* = 11.285
Senior nurse	209 (49.3)	30.02 ± 4.58	*p* = 0.001	31.70 ± 2.98	*p* = 0.009	19.96 ± 3.09	*p* = 0.009	81.68 ± 6.45	*p* < 0.001
Supervisor nurse	140 (33.0)	30.26 ± 4.15		32.10 ± 2.65		20.75 ± 3.05		83.11 ± 5.91	
Associate chief nurse or above	25 (5.9)	32.04 ± 5.59		33.16 ± 1.72		21.40 ± 2.96		86.60 ± 6.89	
Work experiences of ICU (years)									
≤5	142 (33.5)	28.69 ± 5.76	*F* = 9.861	31.66 ± 3.31	*F* = 0.720	19.18 ± 3.63	*F* = 12.489	79.54 ± 7.69	*F* = 16.915
6–10	137 (32.3)	29.69 ± 3.54	*p* = <0.001	31.79 ± 3.17	*p* = 0.540	20.23 ± 2.71	*p* = <0.001	81.70 ± 5.73	*p* = <0.001
11–20	117 (27.6)	31.16 ± 3.83		31.79 ± 3.40		21.18 ± 2.42		84.13 ± 6.12	
≥21	28 (6.6)	32.68 ± 4.89		32.64 ± 2.38		21.93 ± 3.29		87.25 ± 5.65	
Position									
Nurse	370 (87.3)	29.81 ± 4.75	*t* = 0.663	31.83 ± 3.14	*t* = 1.496	20.19 ± 3.13	*t* = 0.011	81.83 ± 6.89	*t* = 0.013
Head nurses	54 (12.7)	31.00 ± 4.23	*p* = 0.416	31.59 ± 3.86	*p* = 0.222	20.69 ± 3.20	*p* = 0.917	83.28 ± 6.95	*p* = 0.908
Training on intravenous medication									
Yes	298 (70.3)	30.84 ± 3.93	*t* = 6.167	31.88 ± 2.92	*t* = 0.757	20.50 ± 2.94	*t* = 2.547	83.22 ± 6.02	*t* = 5.734
No	126 (29.7)	27.88 ± 5.65	*p* < 0.001	31.62 ± 3.88	*p* = 0.450	19.66 ± 3.51	*p* = 0.011	79.16 ± 7.98	*p* < 0.001
**Chinese Calling Scale**	—	—	*r* = 0.305	—	*r* = 0.101	—	*r* = 0.275	—	*r* = 0.380
38.06 ± 7.15			*p* < 0.01		*p* = 0.038		*p* < 0.001		*p* < 0.01

SD, standard deviation.

### ICU nurses’ knowledge, attitudes and practices scores of MEs

Table 2 outlines ICU nurses’ knowledge, attitude, practice, and total scores regarding intravenous MEs prevention, alongside their level distributions. For the knowledge dimension, the mean was 29.96 ± 4.70, and the proportions were 53.8, 35.6, and 10.6% for good, moderate, and poor levels, respectively. Attitude scored 31.80 ± 3.23, where 84.4, 14.9, and 0.7% were categorized as good, moderate, and poor, respectively. Practice had a mean of 20.25 ± 3.14, the percentages were 66.3, 27.4, and 6.3% for good, moderate, and poor levels, respectively. The total KAP score averaged 82.01 ± 6.91, with 70.5% good, 28.8% moderate, and 0.7% poor. The detailed scoring results are presented in Tables 2, 3.

**Table 2:** Knowledge-attitude-practice of intravenous MEs among ICU nurses.

Variables	Mean ± SD (min, max)	Levels	*n* (%)
Knowledge	29.96 ± 4.70 (8–40)	Good (32–40)	228 (53.8)
		Moderate (24–31)	151 (35.6)
		Poor (8–23)	45 (10.6)
Attitude	31.80 ± 3.23 (13–35)	Good (28–35)	358 (84.4)
		Moderate (21–27)	63 (14.9)
		Poor (13–20)	3 (0.7)
Practice	20.25 ± 3.14 (10–25)	Good (20–25)	281 (66.3)
		Moderate (15–19)	116 (27.4)
		Poor (10–14)	27 (6.3)
Total	82.01 ± 6.91 (49–96)	Good (80–96)	299 (70.5)
		Moderate (60–79)	122 (28.8)
		Poor (20–59)	3 (0.7)

SD, standard deviation.

**Table 3:** Score of ICU nurses on knowledge, attitude, and behavior related to intravenous MEs.

Dimension	Items	Scores (mean ± SD)	Percentage
Knowledge			Agree + strongly agree
	K8. Proper storage of medications contributes can reduce intravenous medication errors	4.08 ± 1.00	63.2%
	K3. Provision of pre-packaged by the pharmacy reduces medication errors risk	3.79 ± 0.96	56.8%
	K4. Availability of informative protocols, posters and brochures in the wards, promotes the decrease of the error risk	3.76 ± 0.89	59.2%
	K2. Computerized provide order entry system reduce errors during the preparation’s phase	3.74 ± 0.92	53.5%
	K7. Workload (double shifts, extra time) contributes to pharmacological therapy errors	3.71 ± 0.98	53.8%
	K5. Assistance of a pharmacist during drug preparation reduces the error risk	3.69 ± 0.90	62.7%
	K1. Dosage calculus of intravenous drug reduces preparation errors	3.66 ± 0.94	75.7%
	K6. Alarm noises and ward emergencies may cause distract ions during drugs preparation and administration	3.52 ± 0.95	54.5%
Attitude			Agree
	A7. Medication errors should be reported in order to become an opportunity to improve the care service	4.66 ± 0.90	85.8%
	A6. Clinical skills about safe management of drug therapy should be regularly evaluated	4.64 ± 0.99	86.6%
	A3. The motivation of the workers can improve their professional performance during the whole medication process	4.55 ± 0.97	80.7%
	A5. Protocols/guidelines/procedure can affect professional behavior, ensuring proper management of therapeutic process	4.53 ± 1.11	82.8%
	A4. For a secure management of the entire managing process of IV drugs, some authoritative guidelines drawn up taking into account the available scientific evidence are necessary	4.53 ± 1.05	81.4%
	A1. Ongoing and specific training on safe management of IV drug could reduce the risk of errors	4.46 ± 1.08	77.6%
	A2. Awareness of the prevention of the errors and management of the clinical risk could reduce the errors during the preparation and administration phases of the drugs	4.44 ± 1.03	75.0%
Practice			Agree + strongly agree
	P1. Hand-washing is necessary before the drug preparation and administration	4.33 ± 0.92	88.0%
	P5. Before administration, it is necessary to perform a double check to verify the right correspondence among prescription, preparation and administration of the IV drug	4.31 ± 0.83	87.5%
	P2. A check of vital signs before and after the vasoactive drug administration (dopamine, dobutamine, nitroglycerine, etc.) reduces complications	3.95 ± 0.96	75.7%
	P4. Following the 8R rule (right patient, right medication, right dose, right route, right time, right documentation, right reason, right response) reduces errors	3.88 ± 0.97	69.6%
	P3. Respecting the speed of infusion of the IV administrated solutions (such as chemotherapy, antibiotics, amines, heparin, etc.) reduces errors	3.78 ± 0.92	67.0%

SD, standard deviation.

### Comparison of KAP scores among ICU nurses with different characteristics

Results of single-factor analysis showed that the knowledge and behavior dimension scores of nurses showed statistically significant differences (*p* < 0.05) across age, education level, professional title, ICU working years, and whether they had received intravenous medication safety management training. Similarly, statistically significant differences (*p* < 0.05) in attitude scores were observed among nurses of different ages, genders, hospital levels, and professional titles. Furthermore, Pearson correlation analysis revealed that career calling was positively correlated with knowledge, attitude, and behavior scores related to MEs (*r* = 0.101–0.380, *p* < 0.05). Details are provided in Table 1.

### The multiple linear regression analysis of MEs scale scores among ICU nurses

Multiple linear regression analysis was conducted with ICU nurses’ KAB-MEQ scores as the dependent variable, incorporating independent variables that demonstrated statistical significance in univariate analysis and Pearson analysis (age, education level, professional title, years of ICU experience, receipt of relevant training, and career calling). The results identified five significant predictors of KAB-MEQ scores: education level, professional title, years of ICU experience, receipt of relevant training, and career calling (Table 4). The final model collectively explained 21.1% of the variance in KAB-MEQ scores (*F* = 10.417, *p* < 0.001).

**Table 4:** The results of multiple linear regression analysis of the factors affecting the total scores of ICU nurses’ KAP toward intravenous MEs.

Variables	*B*	SE	*β*	Statistics	*p*-value
(Constant)	70.828	2.23	—	31.763	<0.001
Age (ref: ≤30)					
31–40	0.803	0.714	0.056	1.124	0.261
41–50	2.055	1.048	0.094	1.962	0.05
≥51	1.825	1.816	0.044	1.005	0.315
Education level (ref: Junior college and below)					
Bachelor’s degree	1.958	0.665	0.133	2.946	0.003
Master’s degree and above	3.952	1.513	0.115	2.612	0.009
Professional title (ref: Nurse)					
Senior nurse	2.024	0.951	0.147	2.128	0.034
Supervisor nurse	2.233	1.061	0.152	2.104	0.036
Associate chief nurse or above	3.295	1.558	0.112	2.115	0.035
Work experiences of ICU (ref: ≥21)					
≤5	−3.751	1.305	−0.257	−2.874	0.004
6–10	−3.176	1.281	−0.215	−2.480	0.014
11–20	−1.496	1.284	−0.097	−1.165	0.245
Training on intravenous medication (ref: Yes)					
No	−2.704	0.642	−0.179	−4.209	<0.001
**Chinese Calling Scale** ^a^	0.281	0.042	0.29	6.752	<0.001

*B*: unstandardized coefficients; *β*: standardized coefficients; ^a^continuous variable; *R* = 0.483; *R*^2^ = 0.233; adjusted *R*^2^ = 0.211; *F* = 10.417; *p* < 0.001.

SE, standardized error; ref, reference.

## Discussion

MEs represent a critical global public health challenge that may occur throughout all stages of pharmaceutical management (42). Existing research has systematically investigated ME epidemiology and evaluated compliance with preventative strategies across diverse healthcare environments, including ecological contexts (43, 44). Given this background, the evaluation of healthcare professionals’ KAP regarding MEs assumes strategic significance in developing evidence-based intervention frameworks to mitigate this worldwide patient safety concern. In the current study, nurses demonstrated inadequate knowledge, positive attitudes, and inactive practices toward the prevention of intravenous MEs. It is essential to implement comprehensive training programs and continuous professional development initiatives to enhance nurses’ knowledge and practices related to intravenous medication safety.

### The contradiction between positive attitudes and inactive practices

The attitude dimension of ICU nurses scored relatively high (31.80 ± 3.23), with 84.4% classified as good level. And even the three lowest-scoring items remained at elevated levels, indicating strong consensus among ICU nurses regarding the critical importance of intravenous MEs management. Specifically, among the attitude items, “Medication errors should be reported to improve care service” and “Clinical skills about safe management of drug therapy should be regularly evaluated” had the top two scores. This finding is consistent with the conclusion of a study conducted in Pakistan (45), 89.6% ICU nurses hold a positive attitude toward the necessity of conducting routine assessments of clinical competencies related to the safe management of pharmacotherapy. Such cross-cultural differences highlight the need for context-specific interventions to improve intravenous MEs management, while the overall positive attitude among ICU nurses offers a solid foundation for implementing such initiatives.

Regarding the practice dimension, ICU nurses had a mean score of 20.25 ± 3.14, and only 66.3% were at the good level, indicating that a considerable proportion of nurses have poor performance in intravenous MEs, and targeted interventions are needed to improve this situation. This finding deviates from prior research conclusions (45, 46), and such differences might arise from systemic factors like the distribution of medical resources and the maturity of training systems. Another study on ICU nurses found that almost all followed basic hand hygiene, and over 97% stuck to recommended infusion rates for intravenous medications and monitored vital signs before and after giving vasoactive drugs (47). However, in our study, only 88.0% of nurses reported adhering to basic hand hygiene practices, and 67.0% considered the infusion rate of administration solutions. The three lowest-scoring items monitoring of vital signs before and after vasoactive medication administration, control of intravenous infusion rates, and adherence to the 8R rules. These behavioral deficiencies, coupled with knowledge gaps, form a vicious cycle that significantly elevates intravenous medication administration risks.

Notably, despite ICU nurses’ highly positive attitude toward intravenous ME management, their practice remain inadequate. These findings align with previous research related to the KAP framework (48, 49), which emphasizes that weakness in any component of the KAP theoretical framework could compromise behavioral outcomes. This suggests that strong beliefs alone cannot ensure effective clinical practices and warns against a false sense of security from single-dimension high scores. To address this paradox, targeted interventions should be implemented. First, integrated KAP training programs should be designed, combining knowledge enhancement with skill-based practice and attitude reinforcement. Second, workflow optimization is crucial-incorporating checklists aligned with the 8R rules, and establishing real-time feedback mechanisms for practice adherence. These interventions aim to bridge the gap between positive attitudes and inadequate practice, ultimately reducing intravenous MEs in ICU settings.

### Insufficient knowledge of intravenous MEs

According to the findings of this study, ICU nurses exhibited relatively inadequate knowledge about the prevention of intravenous MEs. In detail, the score for the nurses’ knowledge dimension was 29.96 ± 4.70, and merely 53.8% were categorized as good level. This suggests that their comprehension of knowledge related to the prevention of intravenous MEs still has considerable potential for enhancement. These results align with the findings of other international investigations. Escrivá Gracia et al. (16) carried out a mixed multi-method study among critical-care nurses, and found that 42.5% of the nurses failed more than half the questions on the test. The average score was 47% and the highest score was 69.2%, which reveals a low level of drug knowledge among these professionals. Knowledge deficiencies emerged in three critical areas: dose calculation error reduction, recognition of pharmacists’ collaborative value, and awareness of environmental distractions, revealing gaps in mathematical calculation skills, interprofessional collaboration cognition, and risk perception, revealing gaps in mathematical calculation skills, interprofessional collaboration cognition, and risk perception. This consistency across studies underscores that insufficient knowledge of intravenous medication error prevention is not an isolated issue in a single region but a common challenge faced by critical-care nursing teams, highlighting the urgent need for targeted knowledge training programs to address these deficiencies.

### Influencing factors

#### Education level

Multivariate linear regression analysis revealed that ICU nurses with higher educational backgrounds achieve higher KAP scores in intravenous MEs prevention. The results of a study by Di Muzio et al. (47) showed that ICU nurses’ education degree was associated with MEs prevention attitudes, but the results of their study showed no correlation between nurses’ education and knowledge or practices scores, which is inconsistent with the results of the present study, which could be the result of using a different scale instrument for nurses. The higher KAP scores among nurses with higher educational backgrounds may be attributed to the more comprehensive and rigorous formal education they typically received during their academic training (50). Such training likely provides deeper theoretical foundations in critical areas like pharmacology, evidence-based practice, and patient safety principles. This enhanced knowledge base better equips them to understand and manage the complexities inherent in intravenous medication administration within the critical care environment. However, the survey of this study found that nurses with junior college and below still accounted for 28.3%% in hospitals. Therefore, clinical managers should focus more on the educational upgrading and re-education of nurses with low education. Based on the research findings, it is recommended to implement the tutorial system to improve the prevention attitude of low-educated nurses to intravenous medication safety, and finally achieve the purpose of promoting their active study.

#### Professional title

Professional title was another significant predictor of nurses’ KAP scores regarding intravenous MEs. A clear gradient effect was observed: compared to the reference group (nurse), senior nurses demonstrated a 2.024-point increase in KAP score, supervisor nurses showed a 2.233-point increase, and associate chief nurses or above exhibited the largest gain of 3.295 points. Nurses with senior professional titles demonstrate enhanced research capabilities and clinical competencies (51), who likely possess not only deeper pharmacological knowledge and sharper clinical judgment honed through years of complex patient care but also heightened awareness of system-level risks and safety protocols. Furthermore, most nurses holding senior and intermediate professional titles are head nurses or above in management (52), where they frequently guide junior nurses, review medication processes, lead quality improvement initiatives, and enforce safety standards within the ICU. This combination of accumulated expertise, leadership duties, and accountability for unit-wide safety practices provides a compelling explanation for their superior KAP performance regarding the prevention of intravenous MEs. Therefore, nurses with higher professional titles should be assigned to lead or participate in intravenous medication management for high-risk patients (e.g., those requiring complex intravenous drug regimens or multiple concurrent infusions) and take charge of formulating and optimizing intravenous medication safety protocols. Meanwhile, they can play a mentoring role in training junior nurses, sharing their expertise and experience in intravenous medication error prevention to comprehensively improve the KAP level of the entire ICU nursing team.

#### ICU work experience

In addition, ICU work experience emerged as another notable predictor of nurses’ KAP performance in intravenous MEs prevention. Nurses with shorter ICU work experience had significantly lower KAP scores compared to those with ≥21 years experience. Nurses with ≤5 years scored 3.751 points lower, and those with 6–10 years scored 3.176 points lower. Consistent with the findings in the study on ICU-acquired weakness, which indicated that years of ICU work experience influenced nurses’ KAP. And the more experience ICU nurses have, the more positive their practice behaviors are to prevent the occurrence of acquired weakness (53). Nursing, as a highly practice-oriented discipline, relies heavily on accumulated clinical experience, which largely determines the quality of care delivery (54). Professional competencies typically develop progressively with years of practice, enabling ICU nurses to better manage complex clinical situations, accurately identify potential medication error risks, and cultivate more robust knowledge, proactive attitudes and positive practices. To translate these findings into practical improvements, first, intravenous medication safety management should be incorporated into the pre-job training for newly recruited ICU nurses. Second, in the information age, it is recommended that young nurses utilize online platforms to participate in online courses or watch online videos to enhance their knowledge reserves. Finally, department managers should proactively arrange for nurses with less seniority to attend training programs for intravenous therapy specialist nurses or academic lectures hosted by experts, so as to broaden their horizons and improve their KAP levels in preventing intravenous MEs.

#### Training on intravenous medication

Furthermore, nurses who have received intravenous medication related training tend to show better performance in KAP toward intravenous MEs. Consistently, the results of a qualitative study by Laaksonen et al. (55) showed that the development of relevant teaching or training programs could improve ICU nurses’ attitudes toward MEs prevention and executive competence. And similar to the influence mechanism of factors like thirst management in surgical patients training on nurses’ competencies (56), the lack of systematic intravenous medication knowledge training among ICU nurses may lead to deficiencies in their cognitive understanding of medication error risks, which in turn hinders the formation of positive attitudes and standardized behaviors. From the perspectives of knowledge, attitude and behavior, intravenous medication-related training can systematically strengthen ICU nurses’ professional knowledge reserves such as drug action mechanisms and compatibility contraindications, deepen their cognition of medication error risks, and thus enhance their sense of responsibility for safe medication. Therefore, professional organizations such as nursing associations and health authorities should formulate standardized national training guidelines for intravenous medication safety in ICU nursing. These guidelines should define the core knowledge, attitudes, and skills required, and promote the development of unified training curricula and certification systems. For example, the Chinese Nursing Association could launch nationwide training programs, collaborating with top-tier hospitals to develop advanced courses on intravenous MEs prevention. Health departments should also mandate regular continuing education credits on intravenous medication safety for ICU nurses. Second, nursing managers in hospitals should comprehensively consider various influencing factors to develop more reasonable and effective training programs. And a group-led model can be implemented in the training, in which a nurse who has participated in the training and has high education is arranged to teach two to three nurses who have not participated in the training and have low education (54). Training methods should be vivid and interesting, such as case study workshops, role-playing, and so on. Finally, provide educational resources including pamphlets or information leaflets, posters, and pamphlets in ICU wards. Posters can be designed as tables-each row presenting details on the preparation and administration of common medications-or as quick-reference guides with step-by-step procedural instructions (27).

#### Career calling

Most intriguingly and importantly, this study represents the first investigation into the impact of career calling on ICU nurses’ KAP regarding intravenous MEs. From the multiple linear regression results, the Chinese Calling Scale, as a continuous variable, shows a significant positive impact on the KAP total score of nurses toward intravenous MEs. This indicates that a stronger sense of career calling can effectively promote the improvement of ICU nurses’ knowledge, attitude, and practice levels regarding intravenous MEs. Career calling is defined as an individual’s genuine aspiration and intense enthusiasm for their profession, without the expectation of extra material or financial benefits (57). Nurses who possess a career calling tend to concentrate more on the significance, worth, and responsibility related to their work, which stimulates their work involvement and serves as an inherent driver for their career advancement (58). Xie et al. (36) revealed that career calling serves as a significant predictor of nurse safety behavior. After controlling for socio-demographic characteristics, it independently accounts for 30.7% of the variation in nurse safety behavior. Nurses with high career calling demonstrate a strong professional identity and a more pronounced professional value orientation (59), leading to higher work engagement. Furthermore, nurses with high levels of career calling tend to take a more proactive approach in seeking support from peers and redesigning their work processes to safeguard the safety and quality of nursing care (60). Thus, ICU nurses with a high sense of career calling are more likely to actively explore professional knowledge, including in-depth research on the prevention, identification, and handling of intravenous MEs, because they regard nursing work as a meaningful career mission rather than just a job. In clinical practice, they will be more attentive to every link of intravenous medication, strictly follow safety protocols, and proactively seek ways to improve medication safety, thus showing better KAP performance. Therefore, managers should focus on ICU nurses with low career calling and provide appropriate professional education. And ICU nurses themselves should establish clear career goals, actively seek and cherish all growth opportunities, and deeply integrate personal development into their nursing careers, so that their sense of career calling becomes a powerful driving force to promote the KAP of intravenous MEs prevention.

### Practical implications

This study reveals that although ICU nurses demonstrated a moderately high overall score (82.01 ± 6.91) in KAP toward intravenous MEs prevention, a paradox exists: positive attitudes (84.4% score rate) contrast with insufficient knowledge (53.8%) and practice (66.3%). Additionally, factors such as education level, professional title, work experience, receipt of intravenous medication training, and career calling significantly influence their KAP. To address these findings, nursing managers, educators, and administrators are recommended to design targeted training programs incorporating case-based scenarios and hands-on practice to bridge the gap between attitude and practical application. When scheduling shifts, a strategic pairing of senior and junior nurses should be implemented to facilitate knowledge transfer and peer support. Strengthening continuing education initiatives to update nurses on the latest evidence-based practices, while integrating career calling development programs to enhance professional identity, can further promote safer practice behaviors. Regular KAP assessments should be established to monitor trends, identify persistent gaps, and tailor intervention strategies-such as refresher courses or one-on-one coaching-for continuous improvement.

### Limitations

This research has certain limitations. First, the cross-sectional design of the study makes it impossible to determine the causal relationship between variables, which may lead to temporal ambiguity. Second, the study relies on self-report questionnaires, which may cause reporting bias. For instance, participants who voluntarily take part in the survey might hold a more positive attitude. Third, although the study analyzed the influencing factors of KAP related to intravenous medication errors among ICU nurses, there may be other potential factors, such as individual working environment and organizational support, that were not considered in the study. Future research can use objective measurement methods to improve the objectivity of the results. Last but not least, another limitation of this study is the combined effect of convenience sampling and the specific geographical scope. By recruiting participants based on their availability rather than random selection, the sample may not be fully representative of all ICU nurses in China. Furthermore, given that the study was conducted within a single country and specific geographical areas, the academic, cultural, and social characteristics of nurses vary across countries and healthcare systems, which further limits the external validity and generalizability of the findings regarding ICU nurses’ KAP toward intravenous MEs prevention. Therefore, future research should expand to other countries and diverse healthcare contexts to conduct similar studies, thereby customizing targeted actions aimed at minimizing MEs in ICU and enhancing the generalizability of relevant interventions.

## Conclusion

This study confirms that ICU nurses show a moderately high overall KAP in preventing intravenous MEs, marked by positive attitudes but notable knowledge and practice gaps. Educational level, professional title, ICU experience, specialized training, and career calling significantly influence KAP level of ICU nurses. Building on these insights, targeted interventions are recommended: (1) Strengthening educational programs to address knowledge deficits in dose calculation and interprofessional collaboration; (2) Integrating career calling cultivation through mentorship and professional value seminars to enhance intrinsic motivation; (3) Implementing structured training modules for junior nurses, prioritizing high-risk behavioral protocols; and (4) Establishing multi-disciplinary quality improvement teams to standardize intravenous medication practices.

## Data Availability

The raw data supporting the conclusions of this article will be made available by the authors, without undue reservation.

## References

[1] Donaldson LJ Kelley ET Dhingra-Kumar N Kieny MP Sheikh A. Medication without harm: WHO’s third global patient safety challenge. Lancet. (2017) 389:1680–1. doi: 10.1016/s0140-6736(17)31047-4, PMID: 28463129

[2] Aronson JK. Medication errors: definitions and classification. Br J Clin Pharmacol. (2009) 67:599–604. doi: 10.1111/j.1365-2125.2009.03415.x, PMID: 19594526 PMC2723196

[3] Manias E Kusljic S Wu A. Interventions to reduce medication errors in adult medical and surgical settings: a systematic review. Ther Adv Drug Saf. (2020) 11:2042098620968309. doi: 10.1177/2042098620968309, PMID: 33240478 PMC7672746

[4] Rababa’h AM Mardini AN Ababneh MA Rababa M Hayajneh M. Medication errors in Jordan: a systematic review. Int J Crit Illn Inj Sci. (2022) 12:106–14. doi: 10.4103/ijciis.ijciis_72_21 35845119 PMC9285130

[5] Castro R Aguiar LB Volpe CRG Silva CMS Silva I Stival MM . Determining medication errors in an adult intensive care unit. Int J Environ Res Public Health. (2023) 20:6788. doi: 10.3390/ijerph20186788 37754646 PMC10531059

[6] Poncette AS Spies C Mosch L Schieler M Weber-Carstens S Krampe H . Clinical requirements of future patient monitoring in the intensive care unit: qualitative study. JMIR Med Inform. (2019) 7:e13064. doi: 10.2196/13064, PMID: 31038467 PMC6658223

[7] Tajari M Ashktorab T Ebadi A Zayeri F. Designing and psychometric evaluation of safe nursing care instrument in intensive care units. BMC Nurs. (2024) 23:629. doi: 10.1186/s12912-024-02322-z, PMID: 39256803 PMC11384708

[8] Latif A Rawat N Pustavoitau A Pronovost PJ Pham JC. National study on the distribution, causes, and consequences of voluntarily reported medication errors between the ICU and non-ICU settings. Crit Care Med. (2013) 41:389–98. doi: 10.1097/CCM.0b013e318274156a, PMID: 23263619

[9] Sada O Melkie A Shibeshi W. Medication prescribing errors in the medical intensive care unit of Tikur Anbessa Specialized Hospital, Addis Ababa, Ethiopia. BMC Res Notes. (2015) 8:448. doi: 10.1186/s13104-015-1435-y, PMID: 26376623 PMC4573494

[10] Cho I Park H Choi YJ Hwang MH Bates DW. Understanding the nature of medication errors in an ICU with a computerized physician order entry system. PLoS One. (2014) 9:e114243. doi: 10.1371/journal.pone.0114243, PMID: 25526059 PMC4272266

[11] Marznaki ZH Emami Zeydi A Ghazanfari MJ Salisu WJ Amiri MM Karkhah S. Medication errors among Iranian intensive care nurses: a systematic review. Iran J Nurs Midwifery Res. (2023) 28:123–31. doi: 10.4103/ijnmr.ijnmr_310_21, PMID: 37332377 PMC10275463

[12] Drews FA Markewitz BA Stoddard GJ Samore MH. Interruptions and delivery of care in the intensive care unit. Hum Factors. (2019) 61:564–76. doi: 10.1177/0018720819838090, PMID: 30945959

[13] Wang W Jin L Zhao X Li Z Han W. Current status and influencing factors of nursing interruption events. Am J Manag Care. (2021) 27:e188–94. doi: 10.37765/ajmc.2021.88667, PMID: 34156222

[14] Ma JH Bai Y Xie DS Yang GF. Factors influencing the interruption of nursing document writing in the intensive care unit: a cross-sectional survey. J Multidiscip Healthc. (2023) 16:419–27. doi: 10.2147/jmdh.S394817, PMID: 36820218 PMC9938661

[15] Arslan S Fidan Ö Şanlialp Zeyrek A Ok D. Intravenous medication errors in the emergency department, knowledge, tendency to make errors and affecting factors: an observational study. Int Emerg Nurs. (2022) 63:101190. doi: 10.1016/j.ienj.2022.101190, PMID: 35809484

[16] Escrivá Gracia J Brage Serrano R Fernández Garrido J. Medication errors and drug knowledge gaps among critical-care nurses: a mixed multi-method study. BMC Health Serv Res. (2019) 19:640. doi: 10.1186/s12913-019-4481-7, PMID: 31492188 PMC6729050

[17] McLeod MC Barber N Franklin BD. Methodological variations and their effects on reported medication administration error rates. BMJ Qual Saf. (2013) 22:278–89. doi: 10.1136/bmjqs-2012-001330, PMID: 23322752

[18] Kilic O Gultekin Y Yazici S. The impact of intravenous fluid therapy on acid-base status of critically ill adults: a Stewart approach-based perspective. Int J Nephrol Renovasc Dis. (2020) 13:219–30. doi: 10.2147/ijnrd.S266864, PMID: 33061531 PMC7534048

[19] Kuitunen S Kärkkäinen K Linden-Lahti C Schepel L Holmström AR. Dose error reduction software in medication safety risk management—optimising the smart infusion pump dosing limits in neonatal intensive care unit prior to implementation. BMC Pediatr. (2022) 22:118. doi: 10.1186/s12887-022-03183-8, PMID: 35255846 PMC8902762

[20] Eltaybani S Abdelwareth M Abou-Zeid NA Ahmed N. Recommendations to prevent nursing errors: content analysis of semi-structured interviews with intensive care unit nurses in a developing country. J Nurs Manag. (2020) 28:690–8. doi: 10.1111/jonm.12985, PMID: 32104934

[21] Lan YH Wang KW Yu S Chen IJ Wu HF Tang FI. Medication errors in pediatric nursing: assessment of nurses’ knowledge and analysis of the consequences of errors. Nurse Educ Today. (2014) 34:821–8. doi: 10.1016/j.nedt.2013.07.019, PMID: 23938094

[22] Giannetta N Dionisi S Stievano A Eltaybani S Abdelgawad ME Katigri MR . Comparison across 12 countries on knowledge, attitude, and behavior scores about medication errors in intensive care units: an international study. Eur Rev Med Pharmacol Sci. (2021) 25:7223–30. doi: 10.26355/eurrev_202112_27415, PMID: 34919221

[23] Alandajani A Khalid B Ng YG Banakhar M. Knowledge and attitudes regarding medication errors among nurses: a cross-sectional study in Major Jeddah hospitals. Nurs Rep. (2022) 12:1023–39. doi: 10.3390/nursrep12040098, PMID: 36548171 PMC9783575

[24] Yang L Peng X Song W Su H Wang C Yang S . The barriers to medication error reporting by nurses and factors associated with it: a cross-sectional study in a tertiary hospital of south-west China. BMJ Open. (2025) 15:e091058. doi: 10.1136/bmjopen-2024-091058, PMID: 40295127 PMC12039014

[25] Giannetta N Katigri MR Azadboni TT Caruso R Liquori G Dionisi S . Knowledge, attitude, and behaviour with regard to medication errors in intravenous therapy: a cross-cultural pilot study. Healthcare. (2023) 11:436. doi: 10.3390/healthcare11030436, PMID: 36767011 PMC9914852

[26] Alomari A Wilson V Solman A Bajorek B Tinsley P. Pediatric nurses’ perceptions of medication safety and medication error: a mixed methods study. Compr Child Adolesc Nurs. (2018) 41:94–110. doi: 10.1080/24694193.2017.1323977, PMID: 28557578

[27] Coelho F Furtado L Mendonça N Soares H Duarte H Costeira C . Predisposing factors to medication errors by nurses and prevention strategies: a scoping review of recent literature. Nurs Rep. (2024) 14:1553–69. doi: 10.3390/nursrep14030117, PMID: 39051353 PMC11270417

[28] Gao L Su S Du N Han Y Wei J Cao M . Medical and non-medical students’ knowledge, attitude and willingness towards the COVID-19 vaccine in China: a cross-sectional online survey. Hum Vaccin Immunother. (2022) 18:2073757. doi: 10.1080/21645515.2022.2073757 35612817 PMC9359383

[29] Mäkelä H Dub T Nuorti JP Sane J. Knowledge, attitudes, and practices towards vector-borne diseases in changing climate in Finland. Epidemiol Infect. (2025) 153:e12. doi: 10.1017/s0950268824001468, PMID: 39812232 PMC11748021

[30] Liu S Zhou H Zhang X Yan Q Zhang H Wu C . Knowledge, attitudes and practices of nurses regarding airway clearance in neurocritical illness patients: a cross-sectional study. J Clin Nurs. (2025) 34:464–72. doi: 10.1111/jocn.17341, PMID: 38951120

[31] Homsi MR Davey-Rothwell MA Alonge O Caniza MA Underwood C. Knowledge, attitudes, and practices of healthcare providers regarding vaccinating children with cancer in Latin America and the Caribbean. Vaccine. (2025) 45:126578. doi: 10.1016/j.vaccine.2024.126578, PMID: 39662210

[32] Chen S Yang Z Xie Z Du Y Wu X Yi L . Translation and validation of the knowledge, attitude, and behavior in medication errors questionnaire. J Nurs Sci. (2024) 39:77–81. doi: 10.3870/j.issn.1001-4152.2024.08.077

[33] Ran J Liu H Yuan Y Yu X Dong T. Linking career exploration, self-reflection, career calling, career adaptability and subjective well-being: a self-regulation theory perspective. Psychol Res Behav Manag. (2023) 16:2805–17. doi: 10.2147/prbm.S420666, PMID: 37521566 PMC10378538

[34] Peng J Zhang J Zheng L Guo H Miao D Fang P. Career calling and job satisfaction in army officers: a multiple mediating model analysis. Psychol Rep. (2020) 123:2459–78. doi: 10.1177/0033294119862990, PMID: 31307282

[35] Chang P-C Xiaoxiao G Wu T. Sense of calling, job crafting, spiritual leadership and work meaningfulness: a moderated mediation model. Leadersh Organ Dev J. (2021) 42:690–704. doi: 10.1108/LODJ-09-2020-0392

[36] Xie L Xie S Yu Y Jing J Shi M Dai L. Career calling and safety behavior among nurses: a cross-sectional study based on latent profile analysis. Front Psychol. (2024) 15:1503051. doi: 10.3389/fpsyg.2024.1503051, PMID: 39886369 PMC11780591

[37] Liu N. The sample size estimation in quantitative nursing research. Chin J Nurs. (2010) 45:378–80. doi: 10.3761/j.issn.0254-1769.2010.04.037

[38] Chang Y Zhang XN Yu F Zhang R Li XD Zhao J . Influence of self-perceived burden on quality of life in patients with urostomy based on structural equation model: the mediating effects of resilience and social support. Biomed Res Int. (2022) 2022:9724751. doi: 10.1155/2022/9724751, PMID: 36479307 PMC9722293

[39] Di Muzio M Tartaglini D De Vito C La Torre G. Validation of a questionnaire for ICU nurses to assess knowledge, attitudes and behaviours towards medication errors. Ann Ig. (2016) 28:113–21. doi: 10.7416/ai.2016.2090, PMID: 27071322

[40] Zhang C Herrmann A Hirschi A Wei J Zhang J. Assessing calling in Chinese college students: development of a measure and its relation to hope. J Career Assess. (2015) 23:582–96. doi: 10.1177/1069072715595804

[41] Zhang Y Kuang D Zhang B Liu Y Ren J Chen L . Association between hopelessness and job burnout among Chinese nurses during the COVID-19 epidemic: the mediating role of career calling and the moderating role of social isolation. Heliyon. (2023) 9:e16898. doi: 10.1016/j.heliyon.2023.e16898, PMID: 37303510 PMC10245282

[42] Jambrina AM Santomà À Rocher A Rams N Cereza G Rius P . Detection and prevention of medication errors by the network of sentinel pharmacies in a Southern European region. J Clin Med. (2022) 12:194. doi: 10.3390/jcm12010194, PMID: 36614995 PMC9821611

[43] Liquori G De Leo A Di Simone E Dionisi S Giannetta N Ganci E . Medication adherence in chronic older patients: an Italian observational study using medication adherence report scale (MARS-5I). Int J Environ Res Public Health. (2022) 19:19. doi: 10.3390/ijerph19095190, PMID: 35564585 PMC9100757

[44] Montagna MT De Giglio O Cristina ML Napoli C Pacifico C Agodi A . Evaluation of Legionella air contamination in healthcare facilities by different sampling methods: an Italian multicenter study. Int J Environ Res Public Health. (2017) 14:670. doi: 10.3390/ijerph14070670, PMID: 28640202 PMC5551108

[45] Ahmad T Sitaruno S Pattharachayakul S Kiddeer M Khan SA. Intravenous medication administration practices: insights into knowledge, attitude, and behavior of intensive care unit (ICU) nurses in Pakistan. BMC Nurs. (2025) 24:765. doi: 10.1186/s12912-025-03262-y, PMID: 40597005 PMC12211392

[46] Hamdan KM Albqoor MA Shaheen AM. Intravenous medication errors among ICU nurses: differences in knowledge attitudes and behavior. Open Nurs J. (2022) 16. doi: 10.2174/18744346-v16-e2206201

[47] Di Muzio M De Vito C Tartaglini D Villari P. Knowledge, behaviours, training and attitudes of nurses during preparation and administration of intravenous medications in intensive care units (ICU). A multicenter Italian study. Appl Nurs Res. (2017) 38:129–33. doi: 10.1016/j.apnr.2017.10.002, PMID: 29241505

[48] Mei A Luan M Li P Chen J Mou K. Knowledge, attitude, and practice of psoriatic arthritis among patients with psoriasis. Front Med. (2024) 11:1382806. doi: 10.3389/fmed.2024.1382806, PMID: 39640973 PMC11617161

[49] Tang N Li H Zhang J Ling H Shi L Zhang H . Nurses’ knowledge, attitude, and practice of low-flow oxygen therapy and humidification. Front Med. (2024) 11:1460079. doi: 10.3389/fmed.2024.1460079, PMID: 39624038 PMC11608967

[50] Sheng Q Chen L Tan Y Zhang S Huang Y He T . Knowledge, attitude and practice related to intra-abdominal pressure measurement among intensive care unit nurses and determinant factors: a regional multicentre cross-sectional study. Nurs Crit Care. (2025) 30:e70035. doi: 10.1111/nicc.70035, PMID: 40207442

[51] Yu M Mi J Zhang C Chen H Luo X. Knowledge, attitude and practice regarding hypoactive delirium among ICU nurses: a nationwide cross-sectional study. Nurse Educ Pract. (2023) 72:103749. doi: 10.1016/j.nepr.2023.103749, PMID: 37660518

[52] Sun S Tao L Yang L Zhang Z. Knowledge, attitude, and practice (KAP) of ICU nurses towards tracheal intubation patients’ postextubation dysphagia: a cross-sectional study. J Nurs Manag. (2024) 2024:9981458. doi: 10.1155/2024/9981458, PMID: 40224852 PMC11918944

[53] Zhao M Qiu A Zhang Z Pan F Gao Y. The knowledge, attitude and behavior of ICU nurses regarding ICU-acquired weakness: a cross-sectional survey. BMC Nurs. (2024) 23:377. doi: 10.1186/s12912-024-01942-9, PMID: 38835021 PMC11149312

[54] Sun XX Chen RB Fang PP Yu R Wang XX Liu JQ . Model construction of factors influencing intensive care unit nurses’ medical device-related pressure injury knowledge, attitude, and practice. Int Wound J. (2023) 20:2582–93. doi: 10.1111/iwj.14129, PMID: 36891887 PMC10410357

[55] Laaksonen R Burch AR Lass J McCarthy S Howlett M Silvari V. Patient safety culture and medication safety in European intensive care units: a focus group study. Eur J Hosp Pharm. (2025) 32:209–19. doi: 10.1136/ejhpharm-2024-004212, PMID: 38811151

[56] Zhu Y Lv J Wang X Yang F Zhang W. Knowledge, attitude and practice regarding thirst management in surgical patients among intensive care unit nurses: a cross-sectional study. Nurs Crit Care. (2025) 30:e13176. doi: 10.1111/nicc.13176, PMID: 39382126

[57] Li F Zhou Y Kuang P. Thriving at work, career calling, and moral distress among nurses. Nurs Ethics. (2024) 31:919–29. doi: 10.1177/09697330231215948, PMID: 38116631

[58] Jiang H Mei Y Wang X Zhao Z Lin B Wang W . Professional calling among nursing students: a latent profile analysis. BMC Nurs. (2023) 22:299. doi: 10.1186/s12912-023-01470-y, PMID: 37660012 PMC10474663

[59] Emerson C. Calling to nursing: concept analysis. ANS Adv Nurs Sci. (2017) 40:384–94. doi: 10.1097/ans.0000000000000185, PMID: 28990965

[60] Kallio H Kangasniemi M Hult M. Registered nurses’ perceptions of having a calling to nursing: a mixed-method study. J Adv Nurs. (2022) 78:1473–82. doi: 10.1111/jan.15157, PMID: 35188282 PMC9306482

